# Microfluidic Systems for Biosensing

**DOI:** 10.3390/s100706623

**Published:** 2010-07-09

**Authors:** Kuo-Kang Liu, Ren-Guei Wu, Yun-Ju Chuang, Hwa Seng Khoo, Shih-Hao Huang, Fan-Gang Tseng

**Affiliations:** 1 School of Engineering, University of Warwick, Coventry CV4 7AL, UK; 2 Department of Engineering and System Science, National Tsing-Hua University, Hsinchu, Taiwan; E-Mails: d927111@oz.nthu.edu.tw (R.-G.W.), d927121@oz.nthu.edu.tw (H.S.K.); 3 Department of Biomedical Engineering, Ming Chuang University, Taoyuan County 333, Taiwan; E-Mail: yunju.chuang@gmail.com (Y.-J.C.); 4 Department of Mechanical and Mechatronic Engineering, National Taiwan Ocean University, Keelung 202-24, Taiwan; E-Mail: shihhao@ntou.edu.tw (S.-H.H.); 5 Division of Mechanics, Research Center for Applied Sciences, Academia Sinica, Taipei, Taiwan; E-Mail: Fangang@ess.nthu.edu.tw (F.-G.T.)

**Keywords:** lab-on-a-chip, Micro total analysis systems (μTAS), droplet-based, microfluidic, MEMS, tissue engineering, stem cell, drug delivery

## Abstract

In the past two decades, Micro Fluidic Systems (MFS) have emerged as a powerful tool for biosensing, particularly in enriching and purifying molecules and cells in biological samples. Compared with conventional sensing techniques, distinctive advantages of using MFS for biomedicine include ultra-high sensitivity, higher throughput, *in-situ* monitoring and lower cost. This review aims to summarize the recent advancements in two major types of micro fluidic systems, continuous and discrete MFS, as well as their biomedical applications. The state-of-the-art of active and passive mechanisms of fluid manipulation for mixing, separation, purification and concentration will also be elaborated. Future trends of using MFS in detection at molecular or cellular level, especially in stem cell therapy, tissue engineering and regenerative medicine, are also prospected.

## 1. Introduction

During the last two decades, the exploration of biological systems from molecules, through cells, to small multicellular organisms has explosively grown based on the advancement in microfluidic system. This enabling technology allows sensing of ever-decreasing sample volumes and target analyte concentrations in ways that are not possible using conventional testing systems. Such technology also has the benefit of scaling the dimensions that enables a range of fundamental features to accompany system miniaturization such as reduced reagent consumption, high temporal resolution due to rapid mixing, high throughput, enhanced analytical performance, less waste, low unit cost, reduced energy consumption, and reduced dimensions when compared to macroscale techniques [1]. It is a powerful tool holding great promise to facilitate novel experiments with unprecedented performance and has already found unique applications in chemical and system biology [2–4], high-throughput biological screening [5], cell analysis and clinical diagnostic [6], as well as point-of-care (POC) ion analysis for biomedical and environmental monitoring [7].

Recently, significant development of bioanalysis and clinical analysis has mainly been driven by the strong demand for fast and reliable results, which are essential for early diagnosis and further medical treatment. Results concerning potential drug targets, vaccine studies and speciation of toxic substances must also be of the highest reliability. These bioanalytical challenges in many cases can be solved using specifically designed and fabricated miniaturized tools called lab-on-a-chip systems or micro total analysis systems (μTAS) [8]. Advances in technology have allowed chemical and biological processes to be integrated on a single platform. Adaptation of these approaches to Lab-on-a-Chip (or μTAS) formats is providing a new kind of research tools for the investigation of biochemistry and life processes.

Since this review article is a special issue mainly focused on the state-of-the-art technological development in UK, we highlights some of the most important and interesting recent developments on microfluidics mainly from UK researchers, complementary with some outstanding research findings from international communities.

## 2. Microfluidic Systems and Components

### 2.1. Systems

**Figure 1.**
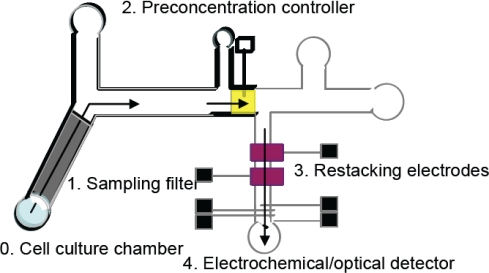
Schematic of one idealized total analysis device showing the various functions on a micro fluidic chip [8].

Microfluidic systems for biosensing normally consist of a set of fluidic operation units that allow different biomolecules to be detected and assayed in an easy and flexible manner. Overall, the chip-based platform which has good integration with micro/nano-fluidic components is capable of sampling, filtration, preconcentration, separation, restacking, and detection for biomolecules. Figure 1 shows a generalized schematic of the types of functional elements used for constructing such a microfluidic chip [9]. Based on their flow type, microfluidic system can be categorized into two main types, continuous and discrete, and their details are reviewed in the following sections (2.1.1 & 2.1.2).

#### 2.1.1. Continuous microfluidic system

Continuous-flow microfluidic operation is a promising approach because it is easy to implement and less sensitive to protein fouling problems. Continuous-flow devices are adequate for many well-defined and simple biochemical applications, and for certain tasks such as chemical separation, but they are less suitable for tasks requiring a high degree of flexibility or complicated fluid manipulations. These closed-channel systems are inherently difficult to integrate and scale because the parameters that govern the flow field vary along the flow path making the fluid flow at any one location dependent on the properties of the entire system. Permanently-etched microstructures also lead to limited reconfigurability and poor fault tolerance capability.

For biosensing or diagnostic applications, the microfluids involved are biomolecules or chemicals derived from biological tissues or body fluids. For the purposes of simple and point-of-care diagnostics, that sample is most likely blood, saliva, or nasal fluid. The preparative steps, including sample collection, metering and filtration, analyte enrichment, labeling and detection, are generally required prior to the diagnostic measurements. Recently advanced microfluidic systems not only provide elegant solutions to relieve the complexity of a biosensing or diagnostic test, but also improve responsive speed and miniaturize the size of analysis equipment [10].

#### 2.1.2. Discrete microfluidic system

Droplet-based microfluidic systems are currently an emerging area of microfluidic research. One of the most popular means is to inject multiple laminar streams of aqueous reagents into an immiscible carrier fluid and therefore to induce flow instability instantly for forming the droplets [11]. There are several distinctive advantages based on droplet-based microfluidic systems. First, the systems promise a new high-throughput technology that enables the generation of microdroplets in excess of several thousand per seconds [12]. In addition, parallel and serial in-vitro compartmentalization is possible with this technology. The reagents are confined inside the droplets in water-in-oil (w/o) emulsions and reagent transport occurs with no dispersion [13]. This unique feature enables chemical reaction indexing, thereby facilitates many chemical reactions in a highly organized manner. Furthermore, fast mixing can occur within minute volumes of microdroplets (nanoliter to femtoliter range) due to the short diffusion distance and chaotic mixing within droplets with the use of twisting channel geometries by stretching, folding, and reorienting fluid [2]. Another feature is that the variation of the channel dimensions can regulate the droplet volumes and decrease volumes anything up to 10^9^ times compared to the smallest assays in conventional microtiter plates [13]. With a control of flow rate, the reagent concentrations can be modified accordingly [2]. It is the confluence of the aforementioned unique features and the ability to regulate and manipulate the droplet motions to split, merge, and sort that has revolutionized our ability to control fluid/fluid interfaces for use in fields ranging from material processing and biomaterials to chemical biology and nanomedicine.

### 2.2. Components

Microfluidic system normally consists of a micropump, micromixer, valve, separator and concentrator. Among these components, micropumps and micromixers are the key components for microfluidic applications due to their actively functioning capability. The types of micropumps vary widely in terms of design and application but can be generally categorized into two main groups: mechanical and non-mechanical pumps. Conventional mechanical micropumps represent smaller versions of macrosized pumps that typically consist of a microchamber, check valves, microchannels and an active diaphragm to induce displacement for liquid transportation. Thermal bimorph, piezoelectric, electrostatic and magnetic forces, as well as shape memory mechanisms, have been utilized to actuate the diaphragm [14–17]. These micropumps are relatively complicated, expensive, typically made by multi-wafer processes and difficult to be integrated with other systems such as integrated circuits (IC) for control and signal processing due to incompatible processes and structures [18–21]. They generally have a large dead volume, leading to excessive waste of biosamples and reagents which are very expensive and precious in biological analysis, especially for forensic investigations. These micropumps typically have moving parts which lead to a high failure rate, low production yield in fabrication and poor reliability in operation. These technologies are based on the manipulation of continuous liquid flow through microfabricated channels. Actuation of liquid flow is implemented either by external pressure sources, external mechanical pumps, integrated mechanical micropumps, or by combinations of capillary forces and electrokinetic mechanisms [22].

#### 2.2.1. Pump

Controlling fluid flow is crucial in microfluidic devices, especially for processing biochemical reactions. Such a process generally relies on active control by mechanical pressure [23,24], electroosmotic force [8], electrowetting [25,26], and electrochemical reaction [27]. These active manipulations enable close control in a rapid and precise manner. Electrokinetic sampling has been widely used for microfluidic chip, especially for microfluidic chip electrophoresis, because the electric field can be easily and precisely applied to the reservoirs on the chip. The popular mechanism used for these active micropumps is electrokinetic force. Based on the mechanism, various micropumps such as dielectrophoresis, asymmetric electric field, electroosmosis and electrophoresis (the latter two are considered as part of the electrohydrodynamic (EHD) phenomena) [28,29] have been developed.

Moving sample fluids and reagents on a biosensing microfluidic device requires developing a pressure difference in the flow path to direct fluid in one direction or another. Miniaturized versions of positive-displacement pump designs such as gear or peristaltic pumps have been proposed for microfluidic applications, but these all require some external power source or repetitive motion to control. It is desirable for fluidic motion in a passive microfluidic system design to be driven by a readily available force such as gravity, capillary action, absorption in porous materials, chemically induced pressures or vacuums (e.g., by a reaction of water with a drying agent), or by vacuum and pressure generated by simple manual action. Wicking and capillary action have been widely used to motivate fluids for POC diagnostics. For example, low cost lateral flow tests demonstrate the elegant and inexpensive use of wicking to drive multiple sample types through all steps of an assay.

One of the simple methods for transporting fluids on microfluidic devices is to apply pressure manually to deflect a diaphragm [30]. Diaphragm membrane pumps have been demonstrated successfully in moving fluid on a microfluidic device. However, it is not easy to control the flow rate in a reproducible way. Zhu *et al.* [31] reported a gravity microfluidic pump for producing constant flow rate. This passive system employs a microchannel and a gravity-driven pump consisting of horizontally oriented reservoirs that supply fluid to the microchannel at a substantially constant rate. The passive device may be useful for numerous microfluidic applications such as cell-size sorting [32]. The pumps have been developed based on osmotic pressure as the actuation mechanism have been used in many drug-delivery applications to deliver medication over a prolonged period of time [33,34]. The advantages of these pumps include simple construction and the absence of moving parts. Another passive system involves controlled evaporation of a liquid into a chamber with an absorption agent flow [35]. As fluid evaporates from the channel, capillary forces induce fluid flowing from reservoir to replace the evaporating fluid. This micropump has advantages of low cost, high reliability and constant flow rate over a long period of time. The major disadvantage of the evaporation micropump is the need to control environmental conditions for constant flow rates and lower flow rates.

The micropumps have also been developed by employing fluid-responsive polymers to deliver fluids [36]. Fluid-responsive polymers swell when exposed to certain environmental conditions, such as changes in moisture, pH, or temperature. One recent fluid-responsive pump consists of an array of responsive polymers that deforms a flexible membrane made from PDMS and produces flow rates [36]. The disadvantage of the pump is the requirement of pressure to inject the buffer solution in order to active the pump.

#### 2.2.2. Valve

The development of valves for microfluidic systems has been progressing rapidly in recent years. The applications of the microvalves include flow regulation, on/off switching, or sealing of biomolecules, micro or nano particles, chemical reagents, oils, water, bubbles, gases, vacuum and many others. Most of them generally can be divided into two major categories: active microvalves and passive microvalves. Most active microvalves mechanically actuate moving parts using magnetic, electric, piezoelectric, thermal or other actuation methods. However, the complexity, high cost and external supporting equipment has largely limited the application of active microvalves. Alternatively, passive microvalves are desirable due to their structure simplicity, easy integration and miniaturization of a system. Passive microvalves can normally be categorized as two categories as with and without moving parts. The passive microvalves with moving parts, also called check valves, are incorporated in inlets and outlets of micropumps as mechanical moving parts, such as flaps [37], membranes [38–40], spherical balls [41] or mobile structures [42]. The passive valves with moving parts only open to forward pressure, excising diode-like characteristics. The one-way behavior of check valves significantly affects the pumping performance of a reciprocal displacement micropump. Leakage in the check valves reduces backpressure and pumping rate in the micropump. The passive microvalves without moving parts; e.g., using nozzle [43,44], diffuser [45–47] or Tesla [48,49] elements, have been widely used in inlets and outlets of micropumps.

Another method to control fluid flow, taking advantage of the large surface-to-volume ratio in microfluidic systems, is the passive capillary microvalve which utilizes the geometries or the surface wetting properties in the microchannels [50–54]. The passive microvalves using capillary effects are useful for passive biosensing microfluidics since autonomous and spontaneous valving can be realized due to the geometry [50–52] and surface wettability properties [53,54] of the microchannels. These passive capillary valves are used preferably to block and pass fluidic flows for avoiding the valve-actuation-induced interference with biofluids due to the actuation energy of microvalves.

#### 2.2.3. Mixer

Mixing is a physical process to achieve homogeneity of the different components involved in the certain process. In some cases, the mixing will be the rate determining step when the mixing time is in the same order or longer than the molecular reaction time. Because the fluid streams mainly appear naturally as laminar flow on a chip, the mixing will mainly depend on molecule diffusion. Mixing small amounts of reagents and samples in microfluidic channels or structures is a challenging task. Likewise, mixing in passive micromixers relies mainly on molecular diffusion and chaotic advection. To speed mixing process, the T-mixer or Y-mixer which consists of the inlets converging into a long microchannel has been developed as a simple and effective solution [55–57]. Other methods for fast mixing have been implemented through reducing the mixing path in a narrow mixing channel [58] and realizing parallel lamination with multiple streams [59,60].

Besides diffusion, advection is another important form of mass transfer in flows with a low Reynolds number. However, advection is often parallel to the main flow direction, and is not useful for the transversal mixing process. The chaotic advection generated by special geometries in the mixing channel can improve mixing significantly. The basic idea is the modification of the channel shape for splitting, stretching, folding and breaking of the flow. The simplest method to get chaotic advection is to insert obstacles or structures in the mixing channel. However, it has been shown that eddies or recirculation cannot be generated in a microchannel, because of its low Reynolds number [61]. The effective method to produce chaotic advection is to modify the wall of mixing channel with ribs, grooves and staggered-herringbone grooves. Johnson *et al.* [62] were the first to investigate this phenomenon. They ablated the grooves on the bottom wall of the channel by laser. This structure allows mixing at a relatively slow velocity of 300 μm/sec. Stroock *et al.* [63] investigate two different groove patterns, slanted groove and staggered. The so-called staggered herringbone mixer can work well at low Reynolds number.

#### 2.2.4. Separator

**Figure 2.**
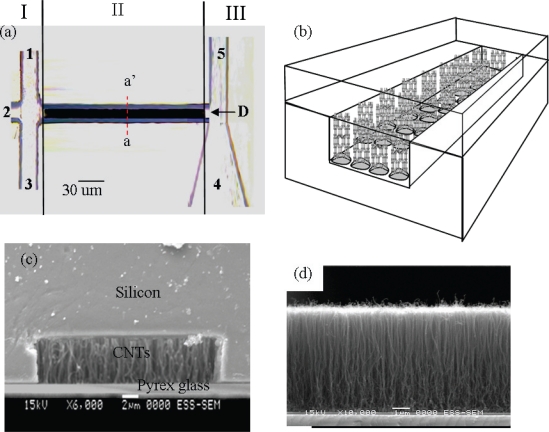
(a) Photographic overhead view of the μCEC chip (dark area in the central channel consisting of MWCNTs array, 1: sample reservoir, 2: eluent buffer/running buffer reservoir, 3: sample waste, 4: eluent buffer waste, 5: running buffer waste, D: detector), (b) The schematic arrangement of MWCNTs in μCEC channels as vertically aligned nanopillars, (c) the SEM image of a cross section (a-a’ plane) of the μCEC channel, (d) The SEM image of vertically-aligned MWCNTs directly grown in microchannel [65].

It is challenging to process complex biological samples without the sophisticated sample preconditioning capabilities for a passive microfluidic system. To answer the challenge, the H-filter system developed by Yager’s group to provide an alternative solution to a conventional porous barrier filter [64]. The H-filter is based upon the parallel laminar flow of two or more miscible streams in contact with each other. The streams do not mix, but species can diffuse from one stream to the other with smaller species diffusing faster than larger ones. The H-filter allows continuous filtration of unwanted components or extraction of desired analytes from fluids without the need for a membrane filter or similar component that requires cleaning or replacement. Wu *et al.* have developed a microcapillary electrochromatography (μCEC) chip for performing a highly efficient separation of double-stranded DNA (dsDNA) fragments through vertically aligned multi-wall carbon nanotubes (MWCNTs) in a microchannel as shown in Figure 2 [65]. This is the first report on the development of a novel stationary nanocolumn by directly growing homogeneous and vertically aligned MWCNTs in microchannels to enable improved analytical performance in capillary electrochromatography. This device incorporated well-arranged nanostructures to replace the non-homogenous traditional packing medium to enhance the phase ratio, reduce the flow resistance, and maintain the laminar flow pattern in a higher efficiency.

#### 2.2.5. Concentrator

**Figure 3.**
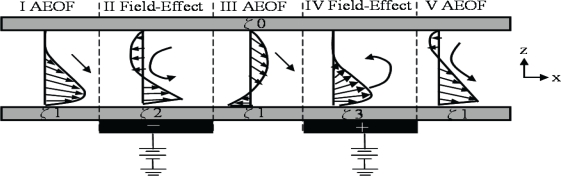

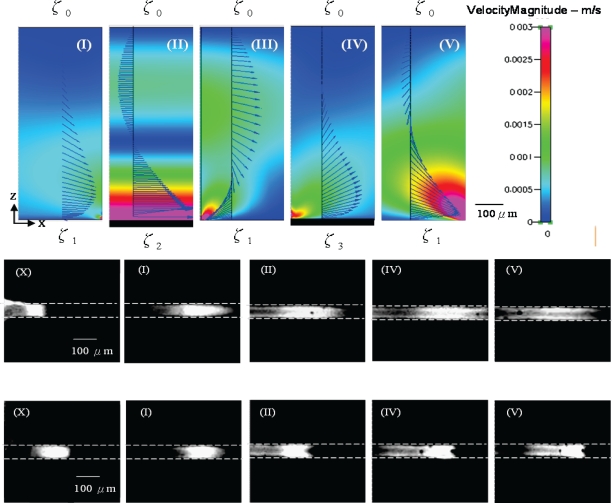
The schematics of (a) flow field evolution for DAEKF in a capillary electrophoresis nanochannel (solid black arrows represent analyte flow direction, and ζ2>ζ1>ζ3>ζ0), (b) detailed flow fields of five different regions for the DAEKF system in a nanochannel. (Region I: a 2-D shear flow, Region II: pulling effect-asymmetric electroosmotic flow (AEOF), Regions IV: pushing effect AEOF). Fluorescence image of Rhodamine B for (c) traditional EOF in a nanochannel, (d) the restacking effect by the DAEKF system in a nanochannel [66].

The concentrator increases concentration of dissolved or dispersed substances under mild conditions to keep activity and viability. They can increase the signal strength of any interesting substance. The most general method is centrifugation. However, this method has not been applied in microfluidic system. Other methods such as chromatography, dielectrophoresis, transverse isoelectric focusing, and ultrasonic trapping have been developed. A novel concentration method employing the dual-asymmetry electrokinetic flow (DAEKF) has been developed by Wu *et al.* [66] for catecholamines concentration and detection in a CEEC nanochannel. The combination of asymmetry EOF and field-effect control was introduced to carry out a two-dimensional gradient shear flow exposed to a downward trailing velocity vector for the generation of a strong downward rotational flow for sample concentration (see Figure 3). However, the method is not suitable for a passive microfluidic system. Sharma *et al.* [67] developed a relatively simple concentrator device based on isothermal evaporation. It is capable of removing 0.8 ml of water per minute at 37 °C, and is also able to concentrate liquids at room temperature at lower evaporation rates. The evaporative concentrator can be used as a stand-alone device or integrated into various processes and analytical instruments, substantially increasing their sensitivity while decreasing processing time.

## 3. Droplet-Based Microfluidics

Due to recent advances in droplet microfluidics that have provided the promise of unique and optimal solutions for the study of genomics, proteomics, cellomics, and metabolomics, many novel device functions and ingenious applications based on this technology have been demonstrated and brought to commercialization. The physics of droplet microfluidics [68], their ways of generating, controlling, and manipulating [69,70], and their applications on chemical kinetics [2], system biology [4], high-throughput biological screening [5], directed evolution of proteins and RNAs and polymerase chain reactions [12,71], and as chemical reactors in microfluidic channels [72] have been extensively reviewed elsewhere in detail. This section highlights the contributions mainly from UK researchers on the physics of droplet microfluidics, the operation principles of droplet manipulations in terms of droplet generations, droplet manipulation methods, droplet fission and merging, mixing in droplets, and droplet sorting, and how they are applied to biomedical applications.

### 3.1. Droplet Generations

Vast amount of femtoliter droplets of typically 1 μm to 100 μm in diameter (about 0.5 fl to 0.5 nl volume) in bulk oil solutions can be easily formed by simply adding the reaction mixture to stirred mineral oil containing surfactants without the need for automation. A volume reduction up to 10^9^ is possible compared to conventional microtiter-plate method and excitingly, this allows a one-step, high throughput production of as many as 10^10^ reactions in a total volume of only 1 ml of emulsion [12]. These tiny droplets can mimic the cellular compartmentalization where molecular evolution takes place inside a cell. The model of compartmentalization inspired Tawfik and Griffiths in the late 1990s from the Centre for Protein Engineering and Laboratory of Molecular Biology at the Medical Research Council, Cambridge to develop the method of linking genotype and phenotype at the molecular level by designing a system for *in vitro* evolution that uses man-made compartments, a technique they termed in-vitro compartmentalization (IVC) [73]. The large excess of droplets, each statistically contains a single gene on average, serve as artificial cells that contain all the ingredients for transcription and translation, and the activity of the resulting RNA or proteins. Each droplet can be used as an independent microreactor. They also applied this IVC method to select DNA catalysts [74] and directed evolution of novel Diels–Alderase ribozymes [75]. The main problem that bulk microdroplet generation encounters is wide distribution of microdroplet size.

Micro and nanopipette delivery is popular among biologist and life science researchers. In addition to the commonly used micropipette in molecular biology as well as medical tests which can dispense between 1 and 1,000 μl of liquid droplet, Ying *et al.* from Cambridge University and Imperial College London developed nanopipettes to employ controlled deposition and local delivery of reagents and biomolecules below nanoliter scale regime based on controlled voltage-driven method for mapping of specific species [76]. The nanopipettes can be easily fabricated from borosilicate capillary glass. Operating in conductance solution (usually physiological buffer), one electrode is placed both in the pipette and the bath to maintain a constant ion current by adjusting the pipette-sample distance under a constant voltage between both electrodes to act as a scanning ion conductance microscopy. By applying an electrical potential to the bath electrode opposite to the charge of the sample in the pipette reservoir, the sample can be driven out from the pipette and absorbed and bound to the nearest surface. Based on this method, the delivery of fluorophore labeled DNA from the pipette was demonstrated and it was found that on application of a voltage pulse, they can get controlled pulses of DNA from the pipette down to the level of just 20 molecules per pulse [77]. This proven method facilitated the controlled insertion of few alpha toxin channels locally into the cell membrane of a cardiac myocyte in a small cluster of cells to study cell-cell communication [76].

Microfluidic systems that exploit flow instabilities between immiscible fluid to generate suspended droplets with the adoption of continuous flow or pseudo continuous flow scheme have been a very popular method for high-throughput droplet generation. In the T-junction method, a droplet can be formed by perpendicularly injecting an aqueous phase into a continuously oil phase. Thorsen *et al.* pioneered the droplet generation method by T-junction [78]. Amici *et al.* from Unilever Corporate Research in Bedford have designed a modified T-junction geometry to produce monodispersed alginate gel droplets by employing two aqueous streams, one acidic and one containing alginate and calcium carbonate and they merge immediately prior to entering a channel where a continuous flow of sunflower oil breaks the flow of the aqueous phase to form microdroplets [79]. Build-up of pressure upstream of the growing drop dictates the dynamics of drop break-up within the device, and the size of the drops was determined by the flow rates of the discontinuous and continuous fluids and independent of the fluid viscosities or their interfacial tensions.

The T-junction channels can also be combined with other operating microfluidic devices for multi-purpose applications. Niu *et al.* from Imperial College London integrated a T-junction channel (with an oil inlet, a sample inlet for effluent from the liquid chromatography capillary, and a droplet outlet channel) and the generated droplets were transferred in sequence to a second device which was composed of channels with pillars for evacuating oil and loading analyte droplets into the second capillary electrophoresis channel to form a fully functional droplet connector for two-dimensional separations in both time and space [80]. The droplet-mediated method could become key components in 2D or multidimensional separations. The stability and monodispersity of the droplet formation based on T-junction can also be used in micro-optical fluidic system in sensing applications due to its wide tenability in the optical characteristics [81]. Chin *et al.* employed a stream of plugs formed by two immiscible liquids, *i.e.*, immersion oil as the carrier liquid and calcium chloride solution as the dispersed liquid at a T-junction, to act as a long-period grating [82]. The liquid grating can be tuned easily based on the flow rates of the liquids, the refractive index, and the index variation of the core layer by using combinations of different liquids to act as a biochemical sensor and an optical tunable filter.

**Figure 4.**
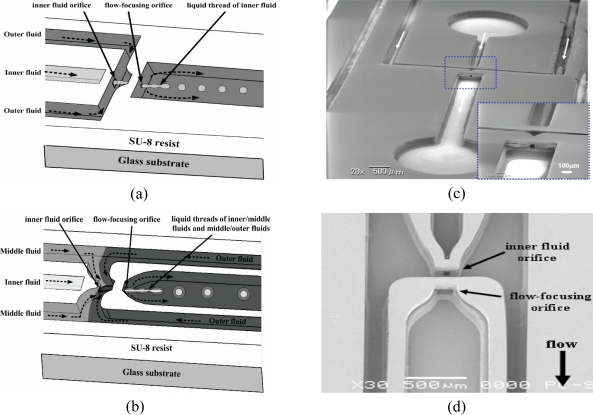
Schematic diagrams of the MEMS-based 3D MFFD using three layers of SU-8 resist for formation of (a) single emulsions and (b) double emulsions. (c) SEM images of the 3D MFFD for formation of single emulsions with inset showing the flow-focusing orifice with dimensions of 50 μm(W) × 50 μm(H). (d) SEM image of the 3D MFFD configuration for formation of double emulsions. The inner fluid orifice measuring 100 μm(W) × 50μm(H) × 100 μm(L) and the flow-focusing orifice measuring 200 μm(W) × 50 μm(H) × 100 μm(L) are coaxial, separated by a distance of 200 μm. The total height of the microchannels is 250 μm. (reprinted with permission from ref. 85, Copyright 2006, IOP).

Numerical simulations have been very helpful in understanding both the intermolecular and hydrodynamic effects in droplet dynamics. Based on the phase-field model and lattice Boltzmann model, Liu and Zhang from University of Strathclyde have recently conducted a comprehensive study on the fluid/fluid interfacial dynamics to understand the mechanisms of droplet formation at T-junction [83]. The capillary number, flow rate ratio, viscosity ratio, and contact angle have been identified as dominating factors during the squeezing and dripping processes. Flow-focusing method is a slightly different method where the dispersed phase is symmetrically sheared and being surrounded by the continuous phase to generate a well-controlled droplet size. Normally, a liquid flows into the middle channel and a second immiscible liquid flows into the two outside channels and they are directed to an orifice [84]. During the flow at the outlet orifice, the pressure and viscous stresses exerted by the outer fluid on the inner fluid is strong enough to form a narrow liquid thread which then breaks into droplets at the vicinity of the orifice, depending on the flow rate and flow rate ratios. To avoid wetting of the channel wall by the dispensed phase, axisymmetric flow-focusing is a promising method to produce monodisperse single or double emulsions for both water-in-oil (W/O) and oil-in-water (O/W) droplets on the same device. Recently, Huang *et al.* demonstrated a three-dimensional (3D) microfluidic flow-focusing device (MFFD) that can produce monodisperse single and double emulsions in a closed and open microfluidic system as shown in Figure 4 [85]. They successfully produced both monodisperse W/O and O/W droplets with a coefficient of variation of less than 4.1 % in the closed channel configuration without any surface modification on the channel wall.

Zourob *et al.* from University of Manchester and University of East Anglia developed a micro-reactor based on a flow-focusing method to produce uniform spherical molecularly imprinted polymer beads for biomedical applications via controlled suspension polymerization in a spiral-shaped microchannel using mineral oil and perfluorocarbon liquid as continuous phases [86]. The produced polymer beads had a coefficient of variation below 2%. It is a one-step continuous flow process with no expensive reagents or equipments are required. In addition, a flow-focusing method to encapsulate cells was employed in a highly sought cell culture lab-on-a-chip developed by Hufnagel *et al.* from University of Cambridge [87]. Based on PDMS/glass, an integrated modular system with seeding, cultivation, manipulation, detachment, collection, and encapsulation of mammalian CHO-K1 cells was fabricated. Encapsulation of CHO-K1 cells into droplets after detachment from the seeding and cultivation device was based on flow-focusing method in a W/O emulsion. A transfection of a reporter gene (encoding green fluorescent protein) was carried to demonstrate that key procedures from the repertoire of cell biology are possible. Low attrition rates (<3%), excellent growth properties in the device for up to 7 days, and the transfection levels (20%) comparable to standard large scale procedures with more than 500 cells could be transfected for CHO-K1 cells were demonstrated.

Performing ultra-high-throughput assays on a very small scale is currently an emerging and powerful format and flow-focusing method is well-suited for compartmentalization of chemical or biological reactions in droplets (typically fL to nL volume) that act as discrete reaction vessels. Courtois *et al.* from University of Cambridge designed an integrated device allowing precisely controlled formation of W/O droplets based on flow-focusing which are stable for at least 6 h in a reservoir, an extended timescale for the study of *in vitro* expression of green fluorescent protein (GFP) [88]. High yields of GFP were obtained and it is possible to perform protein expression from single copies of the DNA template, thereby generating monoclonal droplets.

Dielectrophoretic (DEP) force is used to pull out multiple sessile droplets from a large liquid reservoir [89]. When a voltage is applied to the electrodes, a finger-shaped liquid rivulet with semicircular cross-section is formed which protrudes from the parent droplet reservoir and moved rapidly along the electrodes due to attraction of polarizable fluid to areas of higher electric field intensity. The rivulet stops when it gets to the end of the electrodes and remains in a stable electrohydrostatic equilibrium. When the applied voltage is removed, capillary instability takes over to form sessile droplets within 2 ms. The size and uniformity of the droplets are determined by the magnitude and frequency of the applied voltage. Another important criterion to ensure droplet uniformity is the bumps should be arranged close to the most unstable wavelength predicted by Rayleigh’s cylindrical jet theory. Electrowetting-on-dielectric (EWOD) can also be used to generate droplets. The principle of EWOD is based on the change of free energy on the dielectric surface due to the electric charge accumulation when a voltage is applied, thereby creates a change in wettability on the surface and contact angle of the droplet [90]. All liquid droplet movements are confined between two plates. The device not only can transfer and merge droplets but also generate droplets by cutting an elongated liquid droplet through necking. Through detailed theoretical study, it is found that smaller channel gap, a larger droplet and a larger contact angle change make droplet cutting easier to perform. An excellent report on the comparison between DEP and EWOD can be referred elsewhere [91].

**Figure 5.**
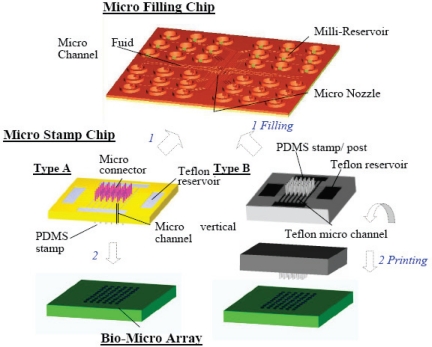
Schematic of microarray system for batch-filling and in parallel printing of multiple proteins. (1) The micro connectors of the micro stamp chips are connected into the nozzles of the micro filling chip, and then the bio-fluids are transferred into the micro stamp simultaneously. (2) After the filling has been completed, those two chips are separated, and then PDMS stamps are used to print in parallel numerous arrays (reprinted with permission from ref. 92, ©2008 IEEE).

It has been understood that capillary force is the prominent force in micro scale regimes. Recently, a novel microcontact printing system capable of printing tens to thousands of biological droplet reagents into an array with batch filling and parallel printing based on the capillary force has been developed as shown in Figure 5 [92]. The system consists of microfilling chip that facilitate the transfer of numerous protein reagents into the microstamp chip by capillary force in seconds. The microstamp chip can then employ the capillary force again to transfer the protein solutions into the corresponding tips and finally come into contact with the substrate for biofluid array printing. More microarray printing devices that have the potential to be expanded to a high throughput system for simultaneously printing large array of biofluid spots for hundred times in minutes, a typical requirement for high throughput disease diagnosis and drug screening, has been demonstrated [93].

### 3.2. Droplet Manipulations

It is generally agreed that moving biological and chemical samples in isolated droplets could provide major advantages over single-phase continuous-flow microfluidic devices as mentioned above. Several methods are currently available to manipulate droplets such as thermocapillary, surface acoustic wave/vibration, surface chemical or morphological gradient, electric field, optoelectrowetting, electrochemical method, magnetic, to name a few. A team from Ecole Polytechnique, University of Dundee and University of St. Andrews recently showed how holographic beam shaping offers greater flexibility for the thermocapillary control of water droplets than Gaussian spots [94]. It can be used to stop droplets in faster flows using less optical intensity when the surface tension variations are created by line patterns instead of single spots. In addition, the variable light patterns can be employed to control droplet routing in a four-way cross microfluidic channel. The multiple droplet storage as well as changing droplet order was demonstrated. The coupling between optical energy and thermocapillarity extends the possibilities of optical manipulation to drop sizes that are not accessible through optical forces alone. A fundamental study on the movement of various sized micro-liter droplets on a surface subjected to temperature gradients can be referred elsewhere [95]. The results show that temperature gradients, the change of dynamic receding/advancing contact angles across the droplets, and the flow fields inside the droplet are the key parameters.

Vibration-based actuation method is also a versatile method to manipulate droplets. Brunet *et al.* from University of Bristol reported an exciting vibration-induced droplet climbing phenomenon against the gravity when the droplet was placed on a vertically vibrating inclined plate [96]. The droplet excised an upward motion when above the threshold in vibration acceleration. The droplet motion is caused by the deformation of the drop as a result of an up or down symmetry breaking induced by the presence of the substrate. This finding allows a droplet to move along an arbitrary path in a plane without special surface treatments or localized forcing. Surface acoustic waves (SAW) were also used to actuate and process very small volumes of fluids (from 50 nL to 100 nL) on the planar surface of a piezoelectric chip [97]. The actuation force is originated from the SAW-mediated internal streaming in the fluid. Recently, a distributed pressure-control scheme has been devised that employs acoustic resonance cavities and rectification structures to translate the frequencies contained in an acoustic signal into separately addressable output pressures that can be used to control liquids in a microfluidic device [98]. This method can be used to perform precise droplet positioning, merging, splitting, and sorting within open microfluidic networks and to generate acoustically tunable liquid gradients.

**Figure 6.**
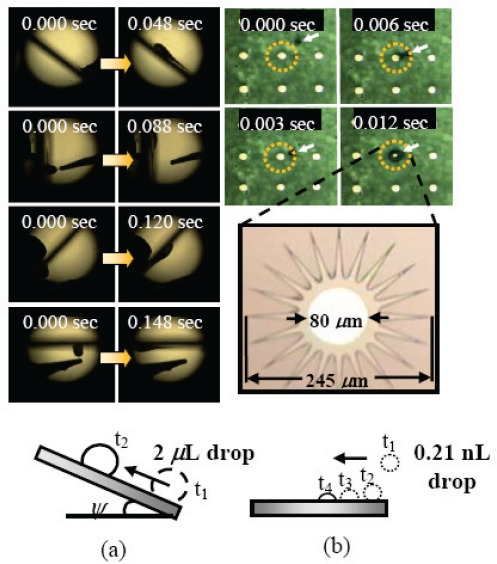
(a) Side view images of a 2 μL surface-ascending water droplet under different inclination angles *θ* (40°, 90°, 130°, and 180°) moving along the gradient with *ψ* = 8° and *L* = 12 mm. (b) A time sequence of top view images of a self-directed subnanoliter water droplet (0.21 nL) moving on a device with an array of circulating wedge-shape gradients. The dashed line indicates the coverage area of the gradients with the detailed dimensions shown in the enlarged view (after photoresist development) of the device. The arrows indicate the position of the droplet of which is transient (reprinted with permission from ref. 101, Copyright 2009, American Institute of Physics).

Surface gradients are passive methods to move droplets where the surface chemical or physical properties gradually change over a given distance. The ways for creating the surface gradients have been extensively reviewed elsewhere [99,100]. An example of a surface gradient based on both the chemical and morphology properties is the wedge-shaped gradients based on low hysteresis nanotextured surfaces recently demonstrated by Khoo and Tseng to facilitate spontaneous and fast motions for a wide range of water droplet volume [101]. A 2 μL droplet velocity as high as 0.5 m/s was successfully achieved. Ascension of water droplets with all-round acclivity and a subnanoliter droplet movement were also successfully demonstrated as shown in Figure 6. It is concluded that the actuation mechanism is based on the combination of surface tension gradient and nanowetting.

Dielectrophoretic (DEP) droplet manipulation can be used to dispense and locate arrays of nanoliter-sized droplets loaded with DNA or protein molecules while separating these particles based on their size [102]. The separation mechanism is based on size-dependent downward directed DEP force exerted on the moving bioparticles resulting from the non-uniform electric field. The separation process is quick, requiring only ∼1 s. The method has also been used to move aqueous droplets for the formation of artificial bilayer lipid membranes (BLMs) [103]. Aghdaei *et al.* from University of Southampton employed dielectrophoretic manipulation method on planar microelectrodes covered by a thin insulator to electrically control aqueous droplets immersed in a lipid-alkane solution. Droplets surrounded by lipid monolayers were brought into contact to form a BLM. This method was used to study membrane protein activity through single channel recording and potentially can be expanded to create programmable BLM arrays and networks. Recently, a light-induced dielectrophoretic droplet manipulation method has become popular. This method enables two-dimensional droplet manipulation on an open chamber with a single-side, featureless photoconductive surface [104]. The principle of actuation is based on electrostatic interaction forces between the electric dipoles induced by the droplet and the light illuminated patterns on the photoconductive surface. Unlike DEP, it does not require electrode wiring and no interconnecting issues. The 2-dimensional droplet transport, merging, mixing, and multidroplet process, for up to 16 droplets in parallel were demonstrated. Its open chamber feature allows easy interfacing and integration with other microfluidic components, such as well and closed channel based droplet devices to increase its versatility for biochemical analyses. A droplet size as small as 5 nL was successfully transported. In another occasion, a Ta_2_O_5_ based dielectric system of an electrowetting-on-dielectric (EWOD) with driving voltage smaller than 15V was employed to propel a silicon swimming robot or pond skating device by a team from University of Tokyo and University of Edinburgh [105]. The trapped air bubbles attached to the hydrophobic surface of the device were moved by EWOD. This low voltage EWOD-driven mechanism is potentially useful for FR power transmission.

### 3.3. Droplet Fusion, Mixing, Sorting, Trapping and Releasing

Many droplet reactions and assays require multiple steps where new reagents are added at defined times, to scale up, start, modify or terminate a reaction. Therefore, device operations such as fusion, mixing, sorting, trapping, and releasing are essential for a fully functional high throughput biochemical device.

For fusion method, Fidalgo *et al.* from University of Cambridge demonstrated a method for droplet fusion based on a surface energy patterning on the walls of a microfluidic device without any active mechanical or electrical parts that allows the fusion of more than two droplets at a single point [106]. Niu *et al.* proposed a novel method to passively merging of aqueous microdroplets within segmented flow microfluidic devices in a controlled manner [107]. Major advantages of this design include the ability to adjust the inter-droplet distance in a facile manner and the ability to selectively merge droplets according to their size or number. The merging mechanism depends on the difference in hydrodynamic resistance of the continuous phase and the surface tension of the discrete phase through the use of pillar structures. The merging process depends on the droplet size, mass flow rate and volume ratio between the droplets and the merging chamber.

Chaotic advection can be employed to rapidly mix multiple reagents isolated in droplets on a microfluidic device by using a combination of turns and straight sections or so-called winding microfluidic channels as long as time-periodic flows are induced [11]. These winding channels create chaotic mixing by folding, stretching and reorienting the fluid volume. The mixing is rapid (sub-millisecond) which allows for an accurate description of fast reaction kinetics. Srisa-Art *et al.* from Imperial College London has applied this method and integrated it with a confocal fluorescence detection system to monitor the real-time streptavidin-biotin binding kinetics [108].

Many complete droplet microfluidic devices have been proposed to conduct complex biochemical experiments. Huebner *et al.* from University of Cambridge, University of London and Imperial College London developed a single-layer poly(dimethylsiloxane) microfluidic structure for the entrapment and release of picoliter-sized W/O droplets in an array format controlled entirely by liquid flow [109]. The array was used to characterize droplet shrinkage, aggregation of encapsulated E. coli cells and enzymatic reactions. The trapped droplets may be recovered from the microfluidic array for further processing. The complete device can generate, store, incubate, monitor and isolate droplets as required. No electric circuits, lasers or valve are needed, therefore it significantly reduces the complexity of fabricating such a device. Mazutis *et al.* from University of Strasbourg and the Laboratory of Molecular Biology at Medical Research Council, Cambridge have developed a droplet microfluidic system in a W/O emulsion which includes droplet generation based on flow-focusing, pairwise droplet fusion based on electro-coalescence method and finally collection and storage [110]. Droplet fusion was performed by electro-coalescence at a rate of ∼3000 fusion events per second. Based on this system, the coupled *in vitro* transcription and translation of genes were followed, after droplet fusion, by an enzymatic assay of the translated CotA protein (laccase). Fidalgo *et al.* from University of Cambridge have integrated mass spectrometry (MS) into a detection scheme for microdroplets created within microfluidic channels [111]. The aqueous droplets are formed in fluorous oil by using flow-focusing. An electric field is applied to coalesce the droplets with the adjacent lateral stream. This design demonstrates a novel microdroplet-continuous microfluidic based device. They have managed to record mass spectra of compounds encapsulated in microdroplets, identify droplets based on their components, and combine fluorescence screening with MS analysis.

Scaling up the microfluidic device networks for microdroplet emulsions is also essential for large production. Recently, Tetradis-Meris *et al.* from Unilever R&D Discover in Sharnbrook has developed a design strategy to scale up microfluidics for producing monodispersed emulsions [112]. Based on this parallelization methodology, a hydrophobic platform containing 180 devices has been successfully demonstrated to produce highly monodispersed W/O emulsions with coefficient of variation ∼5%. The success of this approach can be attributed to correct assessment on the geometric array to be scaled up (ladder-type *versus* tree-type) and the introduction of a drainage manifold architecture which improves the operational conditions. It is anticipated that the number of operating devices can be further increased.

## 4. Microfluidics Devices for Biosensing

### 4.1. Genomic Applications

#### 4.1.1. Microfluidic PCR chip

So far, the most successful commercialization of compartmentalization in Bio-MEMS, *i.e.* water-in-oil droplet, is the emulsion polymerase chain reaction (ePCR). ePCR enables clonal amplification of templates from complex mixtures in a bias-free manner, thus enabling a number of emerging applications such as high-throughput sequencing. However, droplets produced from conventional bulk emulsion techniques are not uniform in size [113]. The non-uniformity of the droplet results in the uncertainty in quantitative readout of experimental data. Another limitation of bulk emulsion droplets is that multistep processing of droplets is difficult. Therefore the application of microfluidic device for the generation of monodispersed droplets will potentially elevate the performance of the existing emulsion PCR technique, through the integration of PCR with other microfluidic operations such as fusion and real-time-analysis. Moreover, the generation of monodispersed droplets facilitates a quantitative analysis, as required for quantitative real-time PCR. The first commercially available platform using microfluidic droplets for various biosensing such as gene expression analysis, drug development, and disease marker detection has been developed by RainDance Technologies [114]. A collection of primer pairs corresponding to selected genomic regions are encapsulated in microfluidic droplets and then merged with droplets containing the genomic DNA and the PCR reaction mixture, followed by off-chip thermal cycling [115].

**Figure 7.**
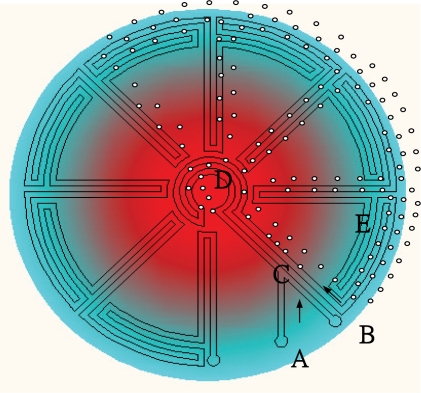
A continuous-flow PCR device where microfluidic droplets move through a temperature gradient toward the radial direction. The device contains an oil inlet (A) that joins an aqueous inlet channels (B) to form droplets (C). The droplets pass through the inner circles in the hot zone (D) to ensure initial denaturation of the template and travel on to the periphery where primer annealing and template extension occur (E). The droplets then flow back to the centre, where the DNA is denatured and a new cycle begins. For illustration, only 7 cycles are demonstrated, but 34 cycles have been achieved in [116].

To enhance the throughput of PCR, a new design device (as shown in Figure 7) for carrying continuous-flow microfluidic droplets in which the reaction mixture passes through zones of alternating temperature corresponding to denaturation, annealing and extension temperatures. For example, microfluidic droplets in a continuous-flow PCR device move through a temperature gradient across the radial direction. The droplets pass through the inner zone with a high temperature to ensure initial denaturation of the template and travel to the outer zone where primer annealing and template extension occur. The droplets then flow back to the centre, where the DNA is denatured and a new thermal cycle begins. Finally, the droplets exit the device after 34 cycles [116]. This new design avoids temperature cycling of the entire device and leads to more rapid heat transfer, and smaller droplets allow higher throughput. The scale-down from milliliter to picolitre droplets in continuous-flow microfluidic PCR may lead to higher sensitivity [117–120]. More recently, microfluidic droplet-based PCR has become a versatile module that can be readily integrated with other unit operations and detection technologies of MEMS, such as fluorescence-based monitoring of PCR products. Amplification in a microfluidic device was monitored online by recording the intensity of a fluorescence resonance energy transfer (FRET) probe for the amplicon [120].

#### 4.1.2. Sequencing and other applications

To achieve *in vitro* directed evolution, the microdroplet boundary acts like the cell wall where the microdroplet compartmentalizes genes and proteins to link the genotype (DNA or RNA) to the phenotype (binding or catalytic activity) [121]. The genes, a single member of a nucleic acid library, are transcribed and translated in microdroplets by an *in vitro* transcription/translation (IVTT) extract. This approach offer a complete *in vitro* system in which the selection environment is not limited to conditions compatible with cell survival (such as pH, temperature or solvents) [121]. Microdroplets containing a desired phenotype are selected by a suitable strategy such as fluorescence detection. Ghadessy *et al.* [122] dispersed the cells into droplets together with primers and dNTPs, and the droplets are subjected to thermal cycling. The polymerase and its gene are released from the cell, allowing self-replication by PCR. Amplified genes are then re-cloned for further selection. Mastrobattista *et al.* [123] dispersed a library of genes into droplets in which the genes are transcribed and translated *in vitro*. Moreover, active enzymes convert a non-fluorescent substrate into a fluorescent product. Fluorescent droplets are separated from non-fluorescent droplets using a fluorescence-dependent sorting. An alternative strategy that takes advantage of sorting by fluorescence is microbead display [124]. Beads carrying one gene of a library, each with an epitope tag, and antibodies against this tag are compartmentalized inside the droplets. The translated proteins can attach to the beads via the epitope tag–antibody interaction. The emulsion is broken down and the beads, displaying multiple copies of the protein, are sorted by fluorescence detection. For understanding the functions of proteins, the microfluidic droplets can also be used to address the effective analysis of enzyme–substrate reactions, protein–protein interactions, and protein modifications. Srisa-Art *et al.* studied the binding kinetics of streptavidin and biotin using FRET between two fluorescent dyes [125] as well as between angiogenin and an anti-angiogenin antibody [126].

### 4.2. Protein Application

#### 4.2.1. Microfluidic chip for separation

Many researchers have exploited the formation of liquid droplets in microfluidic systems to perform a variety of analytical processes due to its distinct advantages with respective to speed, analytical throughput, reagent usage, process control, automation and operational flexibility. In particular, *in vitro* compartmentalization of reactions in water-in-oil droplets combines the necessary ability to carry out large numbers of experiments under controlled conditions with quantitative readout, and has recently advanced towards automation by generating droplets in microfluidic devices.

Generally, liquid droplets can be formed spontaneously through flow instabilities between two immiscible fluid layers. Each droplet is isolated from other droplets therefore each one acts as an individual reaction vessel. More importantly, liquid droplets can be generated at high frequencies (e.g., kHz), meaning that millions of individual reactions can be processed in a single experiment. In addition to droplet formation, the microfluidic technology allows the integration of different unit operations in which droplets can be divided, fused, incubated, analyzed, sorted and broken up [127–129]. Integration of these steps can potentially create a system for demanding biological experimentation. For example, after the droplets have been formed, they can be kept moving in microchannels or captured in traps or reservoirs. The droplets also can be incubated offline and re-injected into the device for further manipulations such as splitting or fusion. To detect the reaction within the droplets, the most frequently used system is based on the readout of fluorescence where the fluorescent droplets can be quantitatively detected and sorted from non-fluorescent droplets [130]. Hollfelder at University of Cambridge and deMello at Imperial College London have done extensive works on droplet-based microfluidics for high-throughput chemistry and biology [131].

#### 4.2.2. Bio-functional polymer particles/microgels assist in concentration

Recently, uniformly micron-sized polymer particles which carry with functional groups are exploited for several biotechnological and biomedical applications, such as affinity chromatography, immobilization technologies, drug delivery, and cell culture [132,133]. Detection, immobilization, and separation of DNA, cells, and proteins require monodisperse particles carrying surface functionalities, e.g., carboxyl, hydroxyl, amine, amide and chloromethyl groups. Polymer particles within the size range of 50 nm to 2 mm can be produced by various manufacturing processes including suspension, emulsion, and dispersion polymerizations [134,135]. However, these methods are usually time-consuming, and provide insufficient control over particle size distribution and particle compositions.

**Figure 8.**
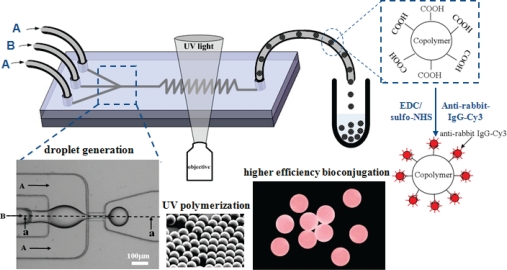
Schematic diagram of MFFD for production of copolymer particles via *in-situ* UV polymerization by co-flow of aqueous (A) and comonomer (B) phases. Typical fluorescent images of the copolymer particles conjugated with IgG-Cy3 for C_AA_ = 40 wt%. Fluid A: DI water + 2 wt% SDS, Fluid B: monomer (ethyleneglycol dimethacrylate, EGDMA) + 0∼40 wt% acrylic acid (AA) + 4wt% photoinitiator (HCPK, 1-hydroxycyclohexyl phenyl ketone) [140].

A microfluidic-based method [136,137] for the rapid continuous *in-situ* photopolymerization of monomer droplets emulsified in a microfluidic flow-focusing device (MFFD) has been proposed and proven to produce extremely monodispersed polymer particles. Typically, the two-dimensional (2D) MFFDs composed by PDMS (polydimethyl siloxane) microchannels on glass substrates were extensively used to produce polymer particles. However, wetting properties associated with both liquids and microchannels for current 2D MFFDs are a vital issue in the generations of monomer droplets. This indicates that the wetting of the channel surfaces, *i.e.*, hydrophobicity and hydrophilicity, has a significant effect on the type of dispersion that can be produced [136]. To avoid wetting of the channel wall by the dispensed phase, axisymmetric flow-focusing device (AFFD) is a promising technique as the inner dispensed phase is surrounded by the continuous phase and never touches the channel wall [138,139]. Huang *et al.* [140] proposed a planar 3D MFFD utilizing PDMS with a simplified fabrication process, which can produce monodispersed copolymer particles carrying surface carboxyl groups in the range of 50∼200 μm prepared through *in-situ* UV polymerization of ethyleneglycol dimethacrylate (EGDMA) with acrylic acid (AA) (Figure 8). High efficiency of bioconjugation on carboxylated copolymer particles was successfully demonstrated by increasing the concentration of AA.

#### 4.2.3. Immunoassays and other specific sensing

Lateral Flow (LF) Immunoassays or Immunochromatographic strips (ICS) or Test strips, as they are called, are one of the most successful point-of-care diagnostics devices, commercially available for detecting various health and environmental threats. Lateral flow (LF) immunosensors which use antibodies as a biosensing element serves as rapid, handy, and disposable tools for point-of-care detection. They also can be regarded as one of the most successful microfluidic platforms for μTAS due to their simplicity, cost and integration with other systems [141–144].

Examples of the applications for LF assays include test kits for detecting pregnancy, drugs of abuse, infectious diseases, cancers, and cardiovascular disorders [145]. The system is normally composed of porous membranes which serve to immobilize biological elements for biosensing and to transport sample reagents by capillary force. The basic operation principle of the strips is passive liquid manipulation through capillary forces within the capillaries of a fleece or a micro-structured channel to control the flow of fluids during immunoassay. The liquid samples are loaded into a sample reservoir from where they penetrate the underlying fleeces or direct capillary filling of the strip from a sample loading point for self testing applications. In general, the test results from LF assays are based on optical detection where fluorescent markers or tagged colored particles or enzymes generate signal upon the completion of specific molecular recognition. The sample fluid with proper flow speed passes through the detection zone to ensure the sample molecules tagged by the marker molecules binding to the immobilized element capture molecules/antibodies. Since the concentration of markers is quite low, they have to be collected in the detection zone to get remarkable signals. By using fluorescent markers, the fluorescence at each discrete detection zone is measured by a fluorometer, a reader with some optical components. In addition, gold or latex particles are used to produce a readable signal. The detection molecules binding with nano gold or latex particles accumulate at the detection zone and the color shows up. The use of tagged color particles can achieve cheap and fast reading of assay results; however, it can only be suitable for readout of binary signal assays, such as pregnancy tests. Besides fluorescent and colorimetric techniques, electrochemical method is also used for detection, e.g., blood glucose.

### 4.3. Cellomics Application

#### 4.3.1. Capillary electrophoresis microchip for cells manipulation and analysis

Cell plays a significant role as the fundamental unit of life and hence high efficient and sensitive detection of the biomolecules or subcellular substance released from single cells are desirable for the studies of physiological processes [146]. The single cell analysis approach not only provides complementary information of complex biological systems but also reveals the actual functional interaction of biomolecules on the cellular and tissue structural basis [147]. In fact, single cell studies are much more complicated and time-consuming than their population counterparts, though the response and interaction of individual cells within a heterogeneous population are difficult to be assessed. Various components (nucleic acids, proteins, carbohydrates) of a cell could be separated into different channels on an appropriate miniaturized platform, and then individually analyzed with the help of the respective individual ‘cellomics’ techniques (details see the Figure 1 of [148]).

There are various assays which have been developed on the capillary microfluidic platform for rapid and accurate sensing of biomarkers in clinical diagnostics and point-of-care applications. In developed countries, interest in moving to a more patient-centric point-of-care /home-testing approach arises and near-patient testing using point-of-care devices has become popular. The cardiovascular disease has become one of the predominant causes of death and disability in all developed countries. Early detection of heart failure or heart attack significantly improves a patient's survival rates and minimizes the permanent damage to tissue and organs. Therefore, reliable, fast and accurate sensing of cardiovascular disease is becoming increasingly important in point-of-care settings. Tweedie *et al.* [149] at Ulster University has developed miniaturized point-of-care sensors for cardiovascular disease markers based on impedimetric sensing of cardiac enzyme capture by an antibody layer immobilized on a planar gold electrode sensor and passive microfluidic delivery. They focused on the detection of cardiac markers of heart attack and acute myocardial infarction (AMI) such as BNP, CK-MB, Myoglobin and Troponin I. The use of impedimetric sensing is advantageous as it is label-free, not requiring the use of any fluorescent reagents. Therefore, it is simpler in operation, and potentially, easier for throughput assay. Passive capillary flow input of patient whole blood, or filtered blood/serum, makes such devices simpler to use, and easier and cheaper to manufacture than other micropumped systems. Another group in UK [150] also proposed a point-of-care diagnostic device for a more reliable and earlier assessment of deep vein thrombosis and related blood clotting conditions. The device measures whole blood concentration of D-dimer, a recognized biomarker of increasing blood clotting activity, through immunochemical biosensor comprising antibody captured onto the surface of impedimetric analysis electrodes. The detection system lies within a disposable liquid handling cartridge, containing a microfluidic system for whole blood handling capabilities and test and data management. Its transfer system is integrated with a base unit.

There are a limited number of commercially available systems for point-of-care diagnostics, for example, Biosite Inc., USA [151]. They developed a rapid and accurate clinical diagnostic platform, containing a disposable cartridge and a reader, for cardiovascular diseases and illegal drugs testing in 16 different immunoassays. The disposable cartridge contains passive microcapillaries to control the mixing of samples with reagents and to control the flow of fluid during immunoassay, and also contains a blood filter to trap blood cells. This platform uses dehydrated fluorescent reagents on-board with optical rather than impedimetric sensing. The high sensitivity fluorescent dyes have following characteristics: (1) can be excited with near infrared wavelengths, and therefore are usable with complex biological samples such as serum, plasma, and whole blood; (2) stable for the shelf period of the product; (3) no overlap between emission and excitation spectrums, so that detection can be performed with inexpensive solid state electronic components.

#### 4.3.2. Nanoparticles assist in subcellular analysis

In recent years, suspension arrays [152], which use self-encoded microcarriers as sensing elements, are attracting increasing interest in the field of drug discovery, gene-expression analysis, and clinical diagnosis [153,154]. Photonic crystals used in suspension arrays have been suggested as a new type of optical spectrum-encoding carrier, whose code is the characteristic reflection peak based on their periodical structure. The code of the reflection peak is very stable, and the fluorescent background is low. These properties render photonic crystals suitable for highly sensitive detection [155].

**Figure 9.**
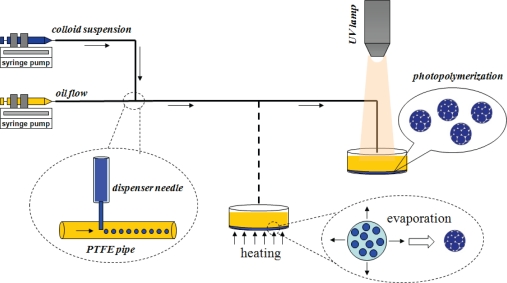
The scheme of the microfluidic device for the fabrication of photonic crystal beads by means of evaporation or UV polymerization to aggregate the colloidal nanoparticles that self-assembled in close-packed structures [156].

Microfluidic devices for the fabrication of colloidal crystal beads have been proposed [156]. In this method, the oil-phase flow and the aqueous-phase flow were simultaneously injected into microchannels by syringe pumps. The aqueous suspension with the colloidal nanoparticles was cut off into droplets by the oil flow. This method can fabricate colloidal crystal beads with controllable size, narrow size distribution, and good repeatability. The generated droplets were then heated or polymerized by UV irradiation to aggregate the colloidal nanoparticles that self-assembled in close-packed structures after solidification (Figure 9). Therefore, the beads showed photonic crystal features, whose reflection peaks were used as the coding elements of biomolecular carriers [157]. Huge coding capacity could be gained by changing the volume fraction of the nanoparticles in the droplets. Gu *et al.* [158] proposed a novel type of microcarrier developed for optical encoding and fluorescence enhancement by depositing semiconductor quantum dots (QDs) on silica colloidal crystals beads (SCCBs). Monodispersed SCCBs were used as the support to deposit different-sized and different-layer QDs, thus leading to wavelength-and-intensity coding. The unique properties of QDs were well suited for multiplexed optical encoding, which could potentially yield a large number of molecular bar codes. The SCCBs possess high porosity and high surface-to-volume ratio (SVR), which can provide stronger detection signals and thus high detection sensitivity. Gu et al. also proposed the encoded porous beads for label-free multiplex of detection of tumor markers by the use of inverse-opaline photonic beads as carriers for suspension arrays [159]. The beads showed large encoding capacity by changing their lattice constant. A label-free multiplex detection of tumor markers, Human CA125, CA19-9, and CEA, which showed great significance in early screening and clinical diagnosis of some tumor diseases including colorectal cancer, gastric cancer, and lung cancer, provides the flexibility and feasibility in emerging clinical applications.

#### 4.3.3. Single cell analysis and droplets compartmentalize cells

From a technical point of view, it is difficult process to perform on biomedical single cells, especially in advanced species. Such analysis calls for significant improvements in terms of cell manipulation, lysis, and separation of cellular constituents, detection sensitivity, and throughput. During the past two decades, a number of novel techniques have been developed for carrying out single cell analysis [160]. Capillary electrophoresis/electrochromatography is one of the excellent techniques for identifying and quantifying the contents of single cells [161]. However, conventional capillary-based techniques lack the ease of high-throughput analysis of single cells due to even smaller sample volumes and masses found in mammalian cells.

The microfluidic devices provide a multiple platform for single cell analysis owing to their unique characteristics to overcome this problem. These devices open the possibility of integrating a variety of cellular operations on the micrometer scale, such as the positioning, trapping or lysis of single cells, as well as detection, analysis and even separation of cellular compounds [162]. Such lab-on-a-chip devices further allow the analysis with improved performance, throughput, parallelization and automation. Lysed cells lose their integrity as well as the likelihood of enabling identification of their specific phenotypes [163]. A goal of modern biology is to understand the molecular mechanisms underlying cellular function. The ability to manipulate and analyze single cells is crucial for this task. The advancement of microengineering has provided biologists with unprecedented opportunities for cell handling and investigation on a cell-by-cell basis [164]. As a result, lab-on-a-chip technologies are emerging as the next revolution in tools for biological discovery. Cell interactions are critical to the function of many organ systems and hence further understanding of cell-material interactions play an important role in the investigation of their physiological traits and functions so as to gain fundamental insight as well as suggest approaches that will allow the manipulation of tissue function *in vitro* for clinical applications [165]. Cell to cell signaling has also been investigated using microfluidic chip based methods [166,167].

Microfluidic droplets can also be used to compartmentalize a number of cell types, such as bacteria [168], yeast [169] and mammalian [170] cells, to study the biological function of a single cell for performing drug screening with high sensitivity of detection. Cells have been shown to remain viable in several oil–surfactant mixtures and device designs. It is possible to incubate the droplets for a long-term storage up to several days offline and re-inject them into a device for analysis [171]. Because the environmental conditions in a droplet can be well controlled, the intracellular components and secretion from cells are constrained within the droplet compartment. The small compartment size creates a high local concentration and leads to high sensitivity of detection. Therefore, cells in droplets can be interrogated by multiple optical methods, such as molecular markers including fluorescent proteins or reporter enzymes [172] for cellular processes, and imaging of cell morphology [173]. In addition, fluorescent life time imaging [174] luminescence detection [175], mass spectrometric analysis [176], and surface enhanced Raman scattering detection [177] have also been demonstrated.

## 5. Prospective

As an enabling technique, microfluidic systems have provided new opportunities for the advancement in several emerging biomedical applications such as tissue engineering, drug delivery/testing, and stem cell therapy.

Tissue engineering aims to cultivate engineered tissues by harvesting cells from patient or donor and then seeding/culturing them on biomimetic scaffolds, fabricated from natural or man-made biomaterials. However, creating 3-D engineered tissue has been limited by how to effectively arrange cells and distribute nutrients into the scaffold during the formation of tissue constructs. To tackle this problem, MFS has recently been used as a tool for fabricating novel “Microfluidic Scaffolds”. As such, embedding microfluidic networks directly within cell-seeded scaffolds can facilitate convective mass transfer for control of the distributions and fluxes of solutes in the bulk of the 3D culture [178]. Microfluidic systems have also been used to develop perfusion-based micro 3-D cell culture platforms for high throughput drug testing [179]. The microfluidic system fabricated based on soft lithography of PDMS and incorporated pneumatic-based pumping culture medium and cell-scaffold loading mechanism to form the cell culture platform has demonstrated to provide a homogenous and steady cell culture environment which will be useful for tissue engineering applications.

Microfluidic system can be easily integrated with microphotonics such as optical trapping to form micro-opto-fluidic system (MOFS) for single cell manipulation [180]. Cultivating single cell in the MOFS can produce homogeneous daughter cells, which have a significant impact on stem cell therapy. The method can potentially apply for the stem cell delivery or the creation of stem cell niche that is to create a microenvironment consisting of various pluripotent cells, appropriate biochemical solutions and mechanical stimuli, e.g., flow shear stress, for fostering stem cells to differentiate into desirable tissues [181]. Recently MFS incorporated with holographic optical tweezers to form a platform which can potentially be used as a reconfigurable force sensor array with piconewton resolution to investigate chemo-mechanical processes [182]. This new technique may provide a powerful tool for multi-cellular manipulation in the cell arrangements and stimuli of engineered tissue [183]. MFS has also been used for the development of *in vitro* physiological systems for studying fundamental biological phenomena for tissue growth [184]. Microfluidic chips have also provided an excellent approach for cell-based screening and detection of different toxicities [185]. This technique can provide a low cost, fast speed, high throughput screening for testing different metabolic responses to drug on a cellular level and hence will be useful for *in-situ* tissue growth monitoring and drug testing. Microfluidic system can uniquely serve as a functional tool for studying cell mechanics which is important for the advancement of drug delivery and tissue engineering. A recent review has comprehensively highlighted its capabilities that are of significance for understanding the mechanical behaviors of cells [186].
